# The nature of memory impairment in multiple sclerosis: understanding different patterns over the course of the disease

**DOI:** 10.3389/fpsyg.2023.1269794

**Published:** 2024-01-16

**Authors:** Jordi Gich, Judit Salavedra-Pont, Clàudia Coll-Martinez, Ester Quintana, Gary Álvarez-Bravo, René Robles-Cedeño, Maria Buxó, Oren Contreras-Rodriguez, Lluís Ramió-Torrentà

**Affiliations:** ^1^Girona Neuroimmunology and Multiple Sclerosis Unit, Neurology Department, Dr. Josep Trueta University Hospital and Santa Caterina Hospital, Girona, Spain; ^2^Neurodegeneration and Neuroinflammation Research Group, Girona Biomedical Research Institute (IDIBGI), Salt, Spain; ^3^Department of Medical Sciences, Faculty of Medicine, University of Girona, Girona, Spain; ^4^Faculty of Psychology and Educational Sciences, University of Girona, Girona, Spain; ^5^Redes de Investigación Cooperativa Orientada a Resultados en Salud (RICORS), Red de Enfermedades inflamatorias (RD21/0002/0063), Instituto de Salud Carlos III, Madrid, Spain; ^6^Department of Statistics, Girona Biomedical Research Institute (IDIBGI), Salt, Spain; ^7^Department of Medical Imaging, Girona Biomedical Research Institute (IDIBGI), Girona, Spain

**Keywords:** multiple sclerosis, memory, retrieval memory deficit, acquisition memory deficit, neuropsychology, cognition

## Abstract

**Introduction:**

Memory deficit is one of the most common and severe cognitive impairments in patients with multiple sclerosis and can greatly affect their quality of life. However, there is currently no agreement as to the nature of memory deficit in multiple sclerosis.

**Methods:**

This cross-sectional study, carried out at the Dr. Josep Trueta and Santa Caterina hospitals in Girona (Spain), was designed to determine the semiology of verbal memory deficit in the different stages of the disease. To this end, a modification of Rey’s verbal auditory test was created by introducing two recognition trials between the five learning trials, thus monitoring what happens in terms of acquisition versus the retrieval of information during the learning phase. Linear regression models were used to evaluate verbal episodic memory performance between-groups adjusting results by age, sex, educational level, and the presence of anxiety and/or depressive symptoms.

**Results:**

133 patients with multiple sclerosis, clinically isolated syndrome, and radiologically isolated syndrome and 55 healthy controls aged 18–65 years were assessed. It was observed that the memory processes of multiple sclerosis patients worsen with the progression of the disease. In this respect, patients in pre-diagnostic phases (radiologically isolated syndrome and clinically isolated syndrome) show no differences in verbal episodic memory compared to the healthy controls. Patients in the inflammatory stage (relapsing–remitting multiple sclerosis) show a previously learned information retrieval deficit, while patients in progressive stages (secondary progressive multiple sclerosis and primary progressive multiple sclerosis) do not even correctly acquire information.

**Discussion:**

These results provide significant information to assist in understanding the nature of memory deficits in multiple sclerosis over the course of the disease. These results are discussed in terms of possible cognitive rehabilitation strategies depending on the evolutive stage and are related to neuropathological mechanisms involved in the progression of the disease.

## 1 Introduction

Multiple sclerosis (MS) is a chronic neurological disease with an autoimmune mechanism that can cause cognitive deficits from its onset. The percentage of involvement increases as the disease progresses (DeLuca et al., 2020). In clinically isolated syndrome (CIS) a 30% rate of cognitive impairment has been reported. In relapsing remitting MS (RRMS) the percentage of patients with cognitive impairment is around 44%, increasing to 79% in the secondary progressive MS (SPMS) and to 90% in primary progressive MS (PPMS) (Ruano et al., 2017).

The most affected cognitive functions are executive functions, information processing speed (IPS) and memory processes (DeLuca et al., 2020). With regard to memory, despite the large number of studies published since the late 1980s, there is no agreement on the nature of the memory deficit suffered by patients with MS (PwMS).

Three hypotheses have been postulated in an attempt to describe the semiology of verbal memory deficits. The first states episodic memory deficits are caused by a disturbance in the process of information retrieval (recall). This position, which was the first to be published (Beatty and Gange, 1977), defends the idea that PwMS perform poorly on free recall tasks, but that their performance increases considerably in recognition tasks (Rao et al., 1993; Coolidge et al., 1996), where they can achieve the same results as the control group (Jennekens-Schinkel et al., 1990; Zakzanis, 2000). The second argues that the main semiological problem of verbal memory in PwMS lies in the difficulty in acquiring new information (encoding-consolidation) (Carroll et al., 1984). To demonstrate this hypothesis, the group led by DeLuca et al. (1994, 1998) used the Selective Reminding Test, increasing the number of free trials until the PwMS were able to learn the list of words up to two consecutive times. This procedure proved to be effective for free memory trials and long-term recognition, with no differences observed between them. Thus, the authors concluded that there is an encoding deficit and that recognition in PwMS is a cognitive process that is preserved when correct encoding of the information has been achieved. These studies have been replicated by researchers from the same group, using other verbal memory tests, namely the logical memory test from the Wechsler Memory Scale-Revised (WMS-R) and a modification of the associated pairs based on Lezak (1995). In this research, although needing many more trials to learn the information, PwMS do not show significant differences in their ability to recall information compared to control subjects when tested at 30 min, 90 min and 1 week after learning (Demaree et al., 2000). According to the authors, these results support the hypothesis put forward by DeLuca et al. (1994, 1998).

The third and final hypothesis that could justify the verbal memory deficit in PwMS focuses on the idea that other cognitive functions – either IPS or working memory (WM), or a combination of the two – might be involved in the outcome of memory processes. Studies have been published supporting both IPS (Litvan et al., 1988) and WM (Thornton et al., 2002). A study attempting to determine which of these two has the greater affect concluded that IPS explains a higher percentage of variance than WM (33% versus 16.2%) as a predictor of performance on the WMS-R logical memory test (Chiaravalloti et al., 2013). Lafosse et al. (2013) found a deficient acquisition may result from demyelination in relevant white matter tracts that reduces encoding efficiency as a result of impaired speed of information processing.

However, methodological considerations may explain the failure to date to achieve clarity in explaining the nature of memory impairment in MS. Some research has included different subtypes of the disease [RRMS and different progressive stages of the disease (SPMS and PPMS)] in the same study group, resulting in global conclusions on memory performance being drawn (Minden et al., 1990; DeLuca et al., 1994; Hulst et al., 2015). On the other hand, other studies have focused on a single subtype of the disease with the result that conclusions can only be drawn regarding the subtype analyzed (Lafosse et al., 2013). Finally, there is research that analyses different clinical forms of the disease separately, but which does not analyze the recognition processes (correct recognition and false positive rate) in depth to discern what type of memory deficit lies behind the memory difficulties of the PwMS (Gaudino et al., 2001). It is also known that non-cognitive symptoms frequently observed in this disease, such as symptoms of anxiety and depression, can have negative effects on cognition, but this is an aspect which is not taken into account when analyzing the results in all studies and so may well contribute to the current lack of consensus on the issue. Much more recent research suggests that the possible effect of symptoms of anxiety and depression on patients’ memory function should be monitored (González Torre et al., 2017).

Given this great disparity in the published results, our objective here has been to describe the nature of the memory deficit in the different evolutionary phases of the disease.

The aim of this project is to describe the nature of memory deficits in MS. Our hypothesis is that PwMS have a retrieval memory deficit of previously learned information. The supporting theoretical framework is based on Tulving’s encoding specificity principle (Tulving, 1983), which suggests that if a stimulus results in the retrieval of a learned item, it is assumed that it has been encoded, whereas if no retrieval occurs, it is assumed that it has not.

Typically, verbal episodic memory tests with word lists assess learning ability, free recall memory after a certain time and finally recognition trials. This assessment procedure has a clear sequential approach. Our working hypothesis is that PwMS have an information retrieval deficit and, moreover, that this deficit is already present from the early stages of learning.

To better understand the semiology of the memory disorder and to be able to demonstrate our hypothesis, we have created a modification of the Rey Auditory Verbal Learning Test (RAVLT) (Strauss et al., 2006, p. 678), which consists of the introduction of two recognition trials between the learning trials. Our conception of the encoding/retrieval processes is that these are interdependent in the acquisition of new information, whereas they can be affected independently, even from the beginning of learning. The introduction of two recognition trials between the learning trials of the RAVLT allows us to evaluate this concept. When patients learn a list of words, we assume that encoding is preserved. However, from the start of learning, the retrieval mechanisms fail, which results in a lower number of retrieved words in the free trials when compared to the control group. The same process happens again and again in each of the free trials.

Thus, if PwMS retrieve previously presented information in recognition trials in the same way as controls, the results would confirm the hypothesis that the memory defect is centered on a retrieval deficit. If, on the other hand, PwMS do not retrieve previously presented information as well as controls, it would point to the memory deficit being in the encoding-consolidation ability.

## 2 Materials and methods

### 2.1 Study design and participants

In this cross-sectional study a total of 133 patients diagnosed with MS and CIS according to McDonald 2010 criteria (Polman et al., 2011), and radiologically isolated syndrome (RIS) according to Okuda criteria (Contentti, 2015) from the Girona Neuroimmunology and Multiple Sclerosis Unit of the Dr. Josep Trueta Hospital (Catalonia, Spain) were included between February 2015 and March 2020. All participants had to have had at least 6 months of disease progression, less than seven points on the Expanded Disability Status Scale (EDSS) (Kurtzke, 1983) and be in a stable neurological condition: without relapses and not having received any corticosteroid treatment in the 30 days prior to inclusion. Patients were classified into three groups according MS subtype. The first group included patients in the preclinical or early stages of the disease (RIS/CIS) and the other two were composed according to Lublin et al.’s (2014) definition: RRMS and progressive multiple sclerosis (PMS).

A total of 55 healthy volunteers made up the control group. Participants with a previous history of substance abuse, those undergoing cognitive rehabilitation and/or participants with head injuries, psychiatric disorders and/or other brain injuries of the central nervous system were excluded.

Study participants had to be between 18 and 65 years old and with at least a basic level of schooling. Standardized protocols, forms, and databases are utilized for data collection to minimize sources of bias.

The study was approved by the Ethics Committee of the Dr. Josep Trueta Hospital (code: 8014), and all participants understood and signed the written informed consent form prior to inclusion.

### 2.2 Procedures

**Figure 1:**
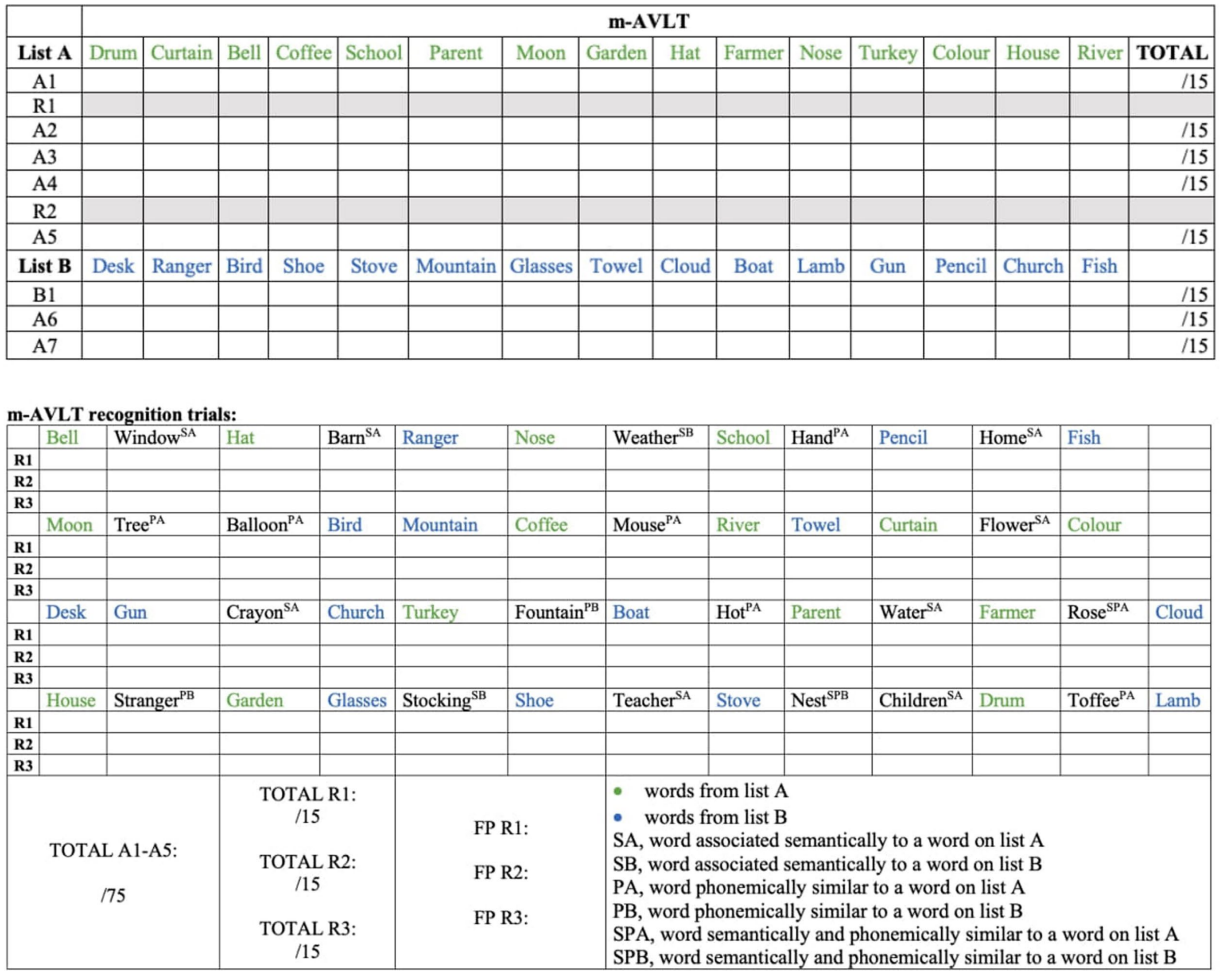
Modified version of the auditory verbal learning test (m-AVLT). m-AVLT, modified Auditory Verbal Learning Test; A1, list A — trial 1; R1, 1st recognition trial; A2, list A — trial 2; A3, list A — trial 3; A4, list A — trial 4; R2, 2nd recognition trial; A5, list A — trial 5; B1, list B — trial 1 (interference list); A6, list A — trial 6 (immediate free recall); A7, list A — trial 7 (delayed free recall); R3, 3rd recognition trial; A1 — A5, sum of the words of the learning trials (A1, A2, A3, A4 and AS). A7, list A — trial 7 (delayed free recall). A, words from list A; S, word with a semantic association to a word on list A or B as indicated; P, word phonemically similar to a word on list A or B; B, words from list B; SP, words both semantically and phonemically similar to a word on the indicated list; FP, false positives.

All participants were assessed at a single time point using an *ad hoc* verbal episodic memory test (m-AVLT) (Figure 1). The memory test administered was a modification of the RAVLT. The original version includes five learning trials of a 15-word list (list A), a 15-word interference list (list B), an immediate and a 20-min free recall trials and, finally, a recognition trial in which the patients must indicate whether the words presented to them were part of the learning list (list A) or not. The modification consisted of adding two recognition trials to the original version; first one between trials one and two, and another between trials four and five.

The modification was considered necessary to analyze in detail what happens to the memory processes (encoding versus retrieval) during the learning phase and after a time interval of 20 min, unlike the traditional version of the test, which only allows what happens to the encoding versus retrieval of the information after the recognition trial to be observed (after the delayed free recall at 20 min).

The IPS and WM, described in the literature as cognitive processes that could influence verbal episodic memory (Litvan et al., 1988; Thornton et al., 2002; Chiaravalloti et al., 2013), were assessed using the symbol digit modalities test (SDMT) and the letter number sequencing (LNS) of the Weschler Adult Intelligence Scale-III (WAIS-III), respectively. Physical disability was assessed by neurologists using EDSS. The following clinical data were also collected: age, sex, years of schooling, clinical form of the disease and time since diagnosis (Table 1).

**Table 1:** Socio-demographic and clinical data by group.

	HC (*n* = 55)	RIS – CIS (*n* = 20)	RRMS (*n* = 66)	PMS (*n* = 47)	*p* value
Women, n (%)	32 (58.18%)	16 (80.00%)	44 (66.67%)	28 (59.57%)	0.31
Men, n (%)	23 (41.82%)	4 (20.00%)	22 (33.33%)	19 (40.43%)	
Age, mean (SD), y	42.82 (11.58)	35.05 (9.09)	43.83 (9.73)	53.51 (7.59)	<0.001
Education level, mean (SD), y	14.96 (3.13)	14.20 (2.93)	12.77 (3.52)	11.11 (3.14)	<0.001
Disease duration^a^, mean (SD), y	–	2.00 (2.05)	10.83 (7.48)	14.21 (9.58)	<0.001
EDSS, mean (SD)	–	1.80 (0.57)	2.02 (1.00)	4.89 (1.42)	<0.001
HADS, mean (SD)	6.76 (5.69)	8.95 (7.02)	10.12 (7.47)	12.26 (5.62)	<0.001

HC, healthy controls; RIS – CIS, radiologically isolated syndrome – clinically isolated syndrome; RRMS, relapsing remitting multiple sclerosis; PMS, progressive multiple sclerosis (secondary and primary progressive multiple sclerosis); EDSS, Expanded Disability Status Scale; HADS, Hospital Anxiety and Depression Scale.

a Time in years between date of neuropsychological assessment to date of diagnosis.

*P* value: statistical significance between patients and control groups according to chi-square test for categorical variables and ANOVA *post-hoc* comparisons with Bonferroni correction for continuous variables. Significant *p* values between groups: age (HC vs. PMS, *p* < 0.001; HC vs. RIS-CIS, *p* = 0.02; RRMS vs. PMS, *p* < 0.001; RRMS vs. RIS-CIS, *p* = 0.003; PMS vs. RIS-CIS, *p* < 0.001), educational level (HC vs. RRMS, *p* = 0.002; HC vs. PMS, *p* < 0.001; RRMS vs. PMS, *p* = 0.05; PMS vs. RIS-CIS, *p* = 0.003), disease duration (RRMS vs. RIS-CIS, *p* < 0.001; PMS vs. RIS-CIS, *p* = 0.007), EDSS (RRMS vs. PMS, *p* < 0.001; PMS vs. RIS-CIS, *p* < 0.001), and HADS (HC vs. RRMS, *p* = 0.04; HC vs. PMS, *p* < 0.001).

#### 2.2.1 Scoring m-AVLT

The results of the different learning trials (m-AVLT A1, A2, A3, A4, A5), and the sum of the five learning trials (m-AVLT A1-A5) were recorded as the total number of correctly recalled words. The same was done for the total number of words recalled from list B (interference list) and for the total number of words recalled in immediate (m-AVLT A6) and delayed free recall at 20 min (m-AVLT A7). However, in the three recognition trials (r1, r2 and r3), where the 15 A-list words were mixed with 35 other distractor words and participants had to identify whether the words belonged to the A-list or not, performance was recorded by the total number of words correctly recognized as A-list words (correct recognition) and the total number of words incorrectly recognized as belonging to the A-list (false positives). Subsequently, the total score of “correct recognition” and “false positive” for each of the three recognition trials was reconverted to rate, and then transformed into a Z-score to analyze the results using the basis of Signal Detection Theory (SDT) (Hautus et al., 2021, p. 3; Russo et al., 2017). SDT allowed us to know the discriminability index (d’) and criterion level (C). The d’, calculated by the formula d’ = Z hits rate – Z false positive rate, is considered the best measure of recognition memory accuracy and indicates the ability of subjects to distinguish previously studied words from distractor words. In this respect, higher d’ values (with a maximum of 4′65) indicate a higher discrimination ability, i.e., more ability to correctly recognize the word as belonging to list A and more ability to reject words that do not belong to the list. C, which indicates the extent to which a subject’s decision criterion is given from a neutral point of view (where words identified as old – previously studied – and new – distracting – would occur with the same frequency), was determined by the formula *C* = −0.5 (Z hits rate + Z false positive rate). In this respect, a negative score would mean a tendency of the subject to say “yes” during recognition trials, while a positive score would mean a tendency to say “no.” Therefore, a *C* = 0 indicates that there is no bias toward either of the two response options (without assessing the level of correctness of those responses).

#### 2.2.2 Confounders of cognitive functioning

Symptoms of depression and anxiety, which may have a negative impact on cognitive functioning, were assessed using the Spanish version of the Hospital Anxiety and Depression Scale (Herrero et al., 2003; Table 1).

### 2.3 Statistical analysis

One-way analysis of variance (ANOVA) for continuous variables with *post-hoc* comparisons with Bonferroni correction and the chi-square test for categorical variables were performed to analyze group differences on all demographic and clinical data. Continuous variables are presented as mean (standard deviation). Categorical variables are presented as frequencies and percentages.

Multiple linear regressions (MLR) were used to evaluate verbal episodic memory performance between-groups adjusting results by age, sex, educational level, and the presence of anxiety and/or depressive symptoms, factors that could explain cognitive differences between groups on their own. Specifically, the sum of the words of the learning trials (m-AVLT A1-A5), the interference list (m-AVLT B1), immediate and delayed free recall (m-AVLT A6 and A7) were analyzed by the total number of words correctly remembered. Scores associated to recognition trials were assessed by d’ and C indexes (see section: Scoring m-AVLT).

Finally, univariate and MLR analysis was performed to examine the predictive relationship between one measure of verbal episodic memory test, specifically the delayed free recall (m-AVLT A7), and measures of IPS (SDMT) and WM (LNS) for each group.

Since relatively few data were missing, we took a complete cases approach to missingness. A value of *p* < 0.05 was considered statistically significant for all analyses. IBM software SPSS^®^ Statistics v.24 was used to perform the statistical analysis.

## 3 Results

**Figure 2:**
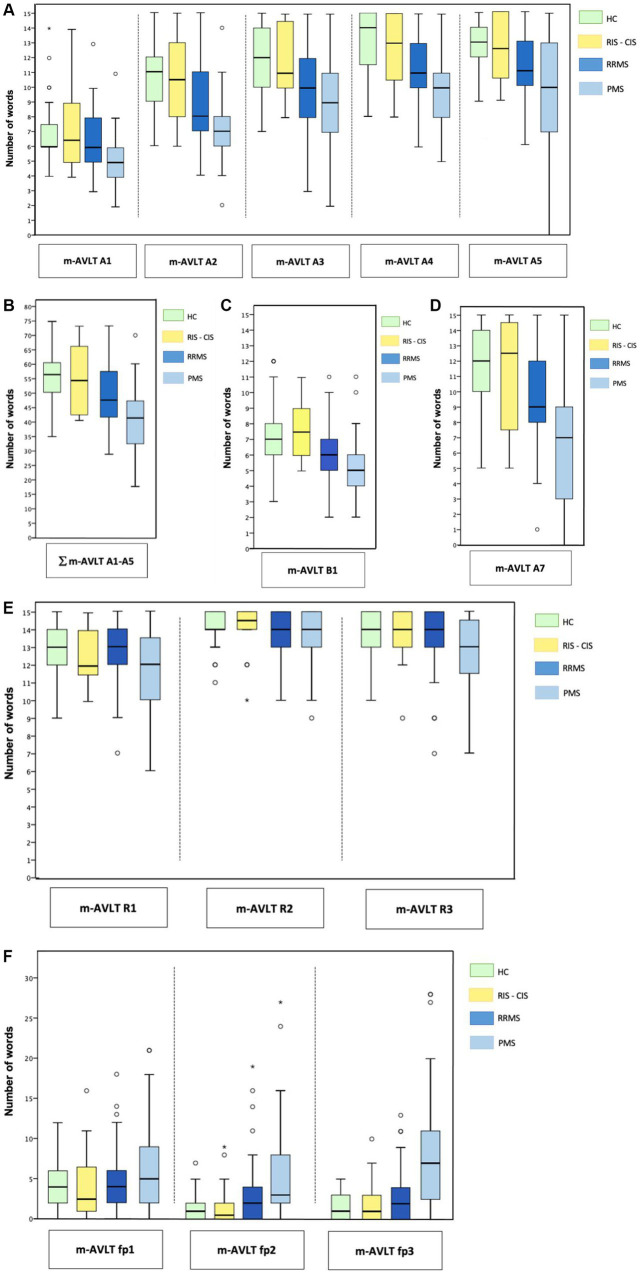
m-AVLT results by groups. Results obtained by each group are presented: **(A)** learning trials; **(B)** number of words recalled during the five learning trials **(C)** interference list; **(D)** delayed free recall; **(E)** recognition trials 1, 2 and 3; **(F)** false positives on recognition trials 1, 2, and 3. Results are presented in line with data shown in Supplementary Table 1. m-AVLT, modified Auditory Verbal Learning Test; A1, list A — trial 1; A2, list A — trial 2; A3, list A — trial 3; A4, list A — trial 4; A5, list A — trial 5; A1 — A5, sum of the words of the learning trials (A1, A2, A3, A4 and A5). B1, List B — trial 1 (interference list); A7, list A — trial 7 (delayed free recall); RIS — CIS, radiologically isolated syndrome — clinically isolated syndrome; RRMS, relapsing remitting multiple sclerosis; PMS, progressive multiple sclerosis (secondary and primary progressive multiple sclerosis).

**Figure 3:**
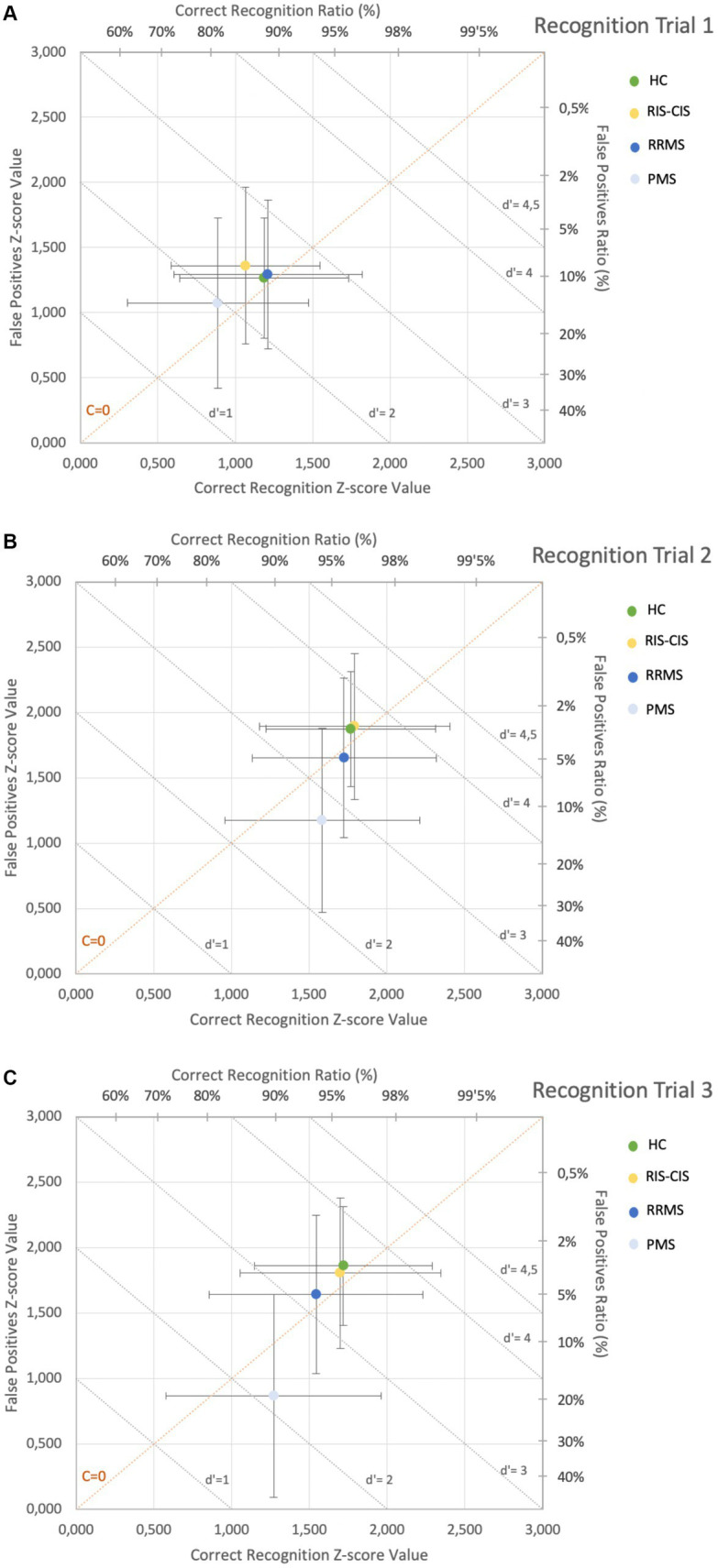
m-AVLT recognition trials by groups. Results obtained by each group during the recognition tasks are presented (results are presented according to data shown in Supplementary Table 2): **(A)** Trial Recognition 1; **(B)** Trial Recognition 2; **(C)** Trial Recognition 3. HC, Healthy Controls; RIS-CIS, Radiologically Isolated Syndrome Clinically Isolated Syndrome; RRMS, Relapsing-Remitting Multiple Sclerosis; PMS, Progressive Multiple Sclerosis (Secondary and Primary Progressive Multiple Sclerosis). Graphs main axis *X* and *Y*: Zscore value of correctly recognized words and false positives, respectively. Secondary axes show the related percentual values of the Zscores for easier interpretation. Gray shaded diagonal lines show the values for the discriminability index (d’), with those values below d’=1 being considered eventful responses and values close to d ’=4 ’5 indicating almost perfect performance. Positive Criterion Level values (located above the dotted diagonal orange line (c=0)) indicate a bias to a NO answer during the recognition task, while the negative *C* values (located below the diagonal orange line) indicate a bias to a YES answer during these recognition tasks. The farther the values are from the diagonal c=0, the more accentuated this bias is.

The study sample consisted of a total of 188 subjects: 20 patients in pre-clinical or early stages of disease (7 RIS and 13 CIS), 66 RRMS, 47 patients in progressive stages of disease (PMS) (27 SPMS and 20 PPMS) and 55 healthy controls (HC). The main socio-demographic and clinical characteristics of the different study groups are set out in Table 1. The neuropsychological results obtained by the different groups in the m-AVLT are shown in Supplementary Tables 1, 2 and are graphically represented in Figures 2, 3.

### 3.1 Neuropsychological comparisons

The results of the MLR comparing performance on the m-AVLT between the disease subtypes versus the HC group are shown in Table 2. No statistically significant differences were observed between RIS-CIS in any of the m-AVLT subtests compared to the results obtained by HC.

**Table 2:** Memory performance between group comparisons.

	RIS – CIS vs. HC			RRMS vs. HC			PMS vs. HC		
	*ß*	95% CI	*p* value	*ß*	95% CI	*p* value	*ß*	95% CI	*p* value
m-AVLT A1	0.018	−0.958, 0.994	0.97	0.035	−0.663, 0.733	0.92	−0.974	−1.804, −0.144	0.02
m-AVLT A2	−0.622	−1.778, 0.534	0.29	−1.135	−1.962, −0.308	0.007	−2.035	−3.018, −1.052	<0.001
m-AVLT A3	−0.676	−1.929, 0.576	0.29	−1.402	−2.297, −0.507	0.002	−2.197	−3.262, −1.132	<0.001
m-AVLT A4	−0.590	−1.769, 0.589	0.33	−1.156	−1.999, −0.313	0.007	−2.150	−3.152, −1.147	<0.001
m-AVLT A5	−0.356	−1.675, 0.963	0.60	−1.061	−2.004, −0.117	0.03	−2.222	−3.344, −1.101	<0.001
m-AVLT A1-A5	−2.234	−7.222, 2.753	0.38	−4.699	−8.266, −1.132	0.01	−9.476	−13.718, −5.235	<0.001
m-AVLT B1	−0.409	−1.449, 0.630	0.44	−1.019	−1.763, −0.276	0.007	−1.666	−2.550, −0.782	<0.001
m-AVLT A6	−0.620	−2.230, 0.989	0.45	−1.002	−2.154, 0.150	0.09	−2.428	−3.800, −1.056	<0.001
m-AVLT A7	−0.878	−2.539, 0.784	0.30	−1.342	−2.530, −0.154	0.03	−3.419	−4.832, −2.007	<0.001
d’1	−0.081	−0.467, 0.306	0.68	0.174	−0.103, 0.450	0.22	−0.218	−0.551, 0.115	0.20
d’2	0.019	−0.390, 0.429	0.93	−0.074	−0.367, 0.219	0.62	−0.524	−0.877, −0.171	0.004
d’3	−0.260	−0.800, 0.280	0.34	−0.181	−0.567, 0.205	0.36	−0.995	−1.454, −0.536	<0.001
C1	0.111	−0.123, 0.345	0.35	−0.014	−0.181, 0.154	0.87	−0.014	−0.215, 0.188	0.90
C2	−0.098	−0.327, 0.131	0.40	−0.097	−0.261, 0.066	0.24	−0.211	−0.408, −0.014	0.04
C3	−0.032	−0.234, 0.171	0.76	−0.035	−0.180, 0.110	0.64	−0.299	−0.470, −0.128	<0.001

Results adjusted by age, educational level, sex, and anxiety-depression.

RIS – CIS, radiologically isolated syndrome – clinically isolated syndrome; HC, healthy controls; RRMS, relapsing remitting multiple sclerosis; PMS, progressive multiple sclerosis (secondary and primary progressive multiple sclerosis); m-AVLT, modified Auditory Verbal Learning Test; A1, list A – trial 1; A2, list A – trial 2; A3, list A – trial 3; A4, list A – trial 4; A5, list A – trial 5; A1–A5, sum of the words of the learning trials (A1, A2, A3, A4 and A5); B1, list B – trial 1 (interference list); A6, list A – trial 6 (immediate free recall). A7, list A – trial 7 (delayed free recall); d’1, discriminability index of 1st recognition trial; d’2, discriminability index of 2nd recognition trial; d’3, discriminability index of 3rd recognition trial; C1, criterion level of 1st recognition trial; C2, criterion level of 2nd recognition trial; C3, criterion level of 3rd recognition trial.

If we compare the m-AVLT performance of RRMS versus HC, we observe significant differences in learning trials A2 (*β* = −1.135, 95% CI −1.962 to −0.308; *p* = 0.007), A3 (*β* = −1.402, 95% CI −2.297 to −0.507; *p* = 0.002), A4 (*β* = −1.156, 95% CI −1.999 to −0.313; *p* = 0.007) and A5 (*β* = −1.061, 95% CI −2.004 to −0.117; *p* = 0.03), as well as in the total number of words learned in the learning phase (m-AVLT A1-A5) (*β* = −4.699, 95% CI −8.266 to −1.132; *p* = 0.01). Differences were also observed in the interference trial (m-AVLT B1) (*β* = −1.019, 95% CI −1.763 to −0.276; *p* = 0.007) and in the 20-min free recall trial (m-AVLT A7) (*β* = −1.342, 95% CI −2.530 to −0.154; *p* = 0.03). Likewise, no significant differences were observed in the performance obtained by RRMS, compared to HC, in any of the three recognition trials (r1, r2 and r3), assessed by the discriminability index (d’1, d’2, d’3) and the criterion level (C1, C2, C3).

Comparing the performance of PMS versus HC we observed differences in all m-AVLT learning trials [A1 (*β* = −0.974, 95% CI −1.804 to −0.144; *p* = 0.02), A2 (*β* = −2.035, 95% CI −3.018 to −1.052; *p* < 0.001), A3 (*β* = −2.197, 95% CI −3.262 to −1.132; *p* < 0.001), A4 (*β* = −2.150, 95% CI −3.152 to −1.147; *p* < 0.001) and A5 (*β* = −2.222, 95% CI −3.344 to −1.101; *p* < 0.001)], as well as in the total number of words learned in the learning phase (m-AVLT A1-A5) (*β* = −9.476, 95% CI −13.718 to −5.235; *p* < 0.001), the interference trial (m-AVLT B1) (*β* = −1.666, 95% CI −2.550 to −0.782; *p* < 0.001), the immediate free recall trial (m-AVLT A6) (*β* = −2.428, 95% CI −3.800 to −1.056; *p* < 0.001) and the 20-min free recall trial (m-AVLT A7) (*β* = −3.419, 95% CI −4.832 to −2.007; *p* < 0.001). Differences were also observed in recognition trials r2 and r3 assessed by the discriminability index [d’2 (*β* = −0.524, 95% CI −0.877 to −0.171; *p* = 0.004) and d’3 (*β* = −0.995, 95% CI −1.454 to −0.536; *p* < 0.001)] and the criterion level [C2 (*β* = −0.211, 95% CI −0.408 to −0.014; *p* = 0.04) and C3 (*β* = −0.299, 95% CI −0.470 to −0.128; *p* < 0.001)].

In the MLR analysis used to examine the predictive relation between delayed free recall (m-AVLT A7) with both IPS and WM for each group, only the PMS group showed a significant amount of variance in the overall model (*R*^2^ = 0.324; *p* = 0.001). For this same group, however, IPS measures (SDMT) as a unique predictive variable accounted for a much higher proportion of variance (*R*^2^ = 0.335; *p* < 0.001) in delayed free recall (m-AVLT A7). Finally, the WM measures (LNS) by itself had the lowest predictive relation (*R*^2^ = 0.109; *p* = 0.03) (Supplementary Table 3).

## 4 Discussion

In the present study we have assessed the verbal episodic memory of PwMS in the different stages of the disease using an *ad hoc* memory test. Our results suggest that the memory performance of PwMS worsens as the disease progresses. Thus, patients in the preclinical (RIS) and early stages of the disease (CIS) do not have long-term memory problems, nor do they show significant differences in the process of encoding-consolidation of information, as they are able to acquire new information, store and retrieve it in the same way as controls do. These findings are in line with those obtained by Ruano et al. (2017), who concluded that cognitive impairment in patients with CIS can be attributed to an impairment of IPS and executive functions rather than to an impairment of other cognitive functions.

In our cohort of RRMS, memory problems are observed in the 20-min free recall trial. However, they have the same ability as the HC to discriminate whether the words in the recognition trial have been previously presented. This difficulty, known as a retrieval memory deficit, indicates that RRMS patients have acquired, encoded-consolidated and stored more stimuli than they have been able to recall unaided. This hallmark observed in our research supports the first hypothesis of memory deficit in MS (Beatty and Gange, 1977; Jennekens-Schinkel et al., 1990; Drake et al., 2006). In this disease group, our working hypothesis is confirmed. In other words, patients acquire the information but are unable to retrieve it spontaneously in free recall trials. Moreover, this deficit is already observed in the early stages of learning. This finding supports the conception of parallel and independent memory processes. Nevertheless, our results are not in line with those published by Fink et al. (2010), as they find a semiological encoding-consolidation deficit in patients with RRMS.

In PMS, greater difficulty is observed in recalling information during the learning phases, in the immediate free recall trial and in the 20-min free recall trial compared to the rest of the study groups. However, the results of recognition tests can be misleading if they are not analyzed in detail. In this respect, although they recognize the words presented above as well as the rest of the groups, the PMS group make a high rate of false positives (saying “yes” to words that were not present in the original list). It is precisely this deficit that clearly stands out in the more severe memory impairment of this group of patients. Taking into account that processes of retrieval by recognition are much easier than retrieval by recall for intact persons as well as for brain damaged patients (Lezak et al., 2012), it is assumed that mechanisms related to consolidation (prior to retrieval) are dysfunctional or become so at this stage of the disease. Along the same lines as our results, Van den Burg et al. (1987) considers that the deficits observed in recognition recall are characteristic of an encoding deficit (in terms of accelerating forgetting). Our findings are inconsistent with studies reporting that recognition processes are preserved in the PMS, which, it should be noted, do not report information on false recognition (Demaree et al., 2000; Gaudino et al., 2001; Müller et al., 2013). In our cohort, the hallmark of this group is the high number of false recognitions. In this respect, several studies have already pointed to clinically similar errors (higher number of errors in recognition trials, confabulation errors, etc.) in PMS (Drake et al., 2006). The nature of the memory deficit observed in this group of patients is consistent with the results described in the literature by DeLuca et al. (1994, 1998) and Demaree et al. (2000). There is a clear deficit in the acquisition of information in PMS, so new information is neither properly acquired nor consolidated.

In the same way that De Meo et al. (2021) and Bergendal et al. (2007) observed a general cognitive worsening over the course of the disease (especially in SPMS), we observed this worsening in the memory semiology, with patients in progressive phases being more affected.

The study by Thornton et al. (2002) concludes that patients with MS have a deficit that affects both the encoding and retrieval of information. In other words, patients with MS access long-term memory correctly through pre-existing associations within the semantic network. However, they access long-term memory less effectively when weakly associated contextual retrieval cues are employed. In this case, MS patients show greater retrieval deficits than controls. Although the study by Thornton et al. (2002) does not differentiate patients on the basis of their clinical stage, the difficulties they describe may correspond to the semiological difficulties we observed in the group of patients in the progressive stages of the disease.

Ultimately, addressing the potential influence of other cognitive functions on the memory performance in MS patients, Thornton et al. (2002) observed a direct relationship between verbal working memory and encoding difficulties in long-term memory in MS patients (this relationship was not observed in the control group of the study).

In recent years, IPS has been found to account for a larger rate of variance in memory processes than WM (Chiaravalloti et al., 2013). In our cohort, IPS has also been found to explain a higher rate of variance than WM in delayed free recall, but this is only seen in the PMS. In contrast to the results observed by Lafosse et al. (2013), where impaired speed information processing influenced memory outcomes in patients in the RRMS clinical phase. Our finding could partially explain the difficulties PMS patients have in acquiring, encoding, and consolidating learned information. These results matching those published in research by DeLuca et al. (1994, 1998), Demaree et al. (2000), and Gaudino et al. (2001). The prolonged presentation of stimuli would probably enhance the ability to acquire new information.

## 5 Conclusion and future directions

To the best of our knowledge, this is the first study to demonstrate that the verbal memory deficit in PwMS evolves throughout the disease stages, from normal in pre-clinical stages of the disease to an encoding deficit in progressive stages, and further into a retrieval deficit in inflammatory stages. The progressively increasing severity of memory deficit may be related to the neuropathological mechanisms involved in each of the developmental stages. Thus, the classic pattern of retrieval deficits can be linked to the phase where neuroinflammation predominates and encoding deficits to the phase where neurodegeneration does.

Our results should also be useful to better plan cognitive rehabilitation techniques and procedures for memory processes at each stage of the disease. Future studies will have to be conducted to determine whether the false positives made by PMS patients are related to memory problems or to inhibitory processes linked to executive functions.

On reflection, and noting the great disparity of results regarding memory and the RRMS, we observe that the inflammatory phase usually lasts many years, and it is probably during this period that the changes in memory semiology occur. Thus, at the beginning of this clinical stage, patients either have no memory deficit, or if they do, it is a retrieval deficit, and as the phase itself evolves over the years, the semiological memory deficit evolves in severity, until reaching an encoding-consolidation deficit that we have observed in the progressive phases of the disease. Future studies with large samples of patients and advanced neuroimaging techniques will attempt to shed light on this probable evolution within the inflammatory phase and its neuroanatomical correlates. In addition, for all other clinical forms of the disease, future lines of research will need to correlate the memory findings described here with structural and functional magnetic resonance imaging.

## Data availability statement

The raw data supporting the conclusions of this article will be made available by the authors, without undue reservation.

## Ethics statement

The studies involving humans were approved by Clinical Research Ethics Committee of the Dr. Josep Trueta Hospital. The studies were conducted in accordance with the local legislation and institutional requirements. The participants provided their written informed consent to participate in this study.

## Author contributions

JG: Formal analysis, Writing – original draft, Writing – review & editing, Conceptualization, Methodology, Project administration, Supervision. JS-P: Formal analysis, Writing – original draft, Writing – review & editing, Data curation, Investigation, Visualization. CC-M: Data curation, Formal analysis, Investigation, Writing – review & editing. EQ: Formal analysis, Writing – review & editing. GÁ-B: Formal analysis, Writing – review & editing. RR-C: Conceptualization, Methodology, Resources, Writing – review & editing. MB: Formal analysis, Writing – review & editing. OC-R: Formal analysis, Supervision, Writing – review & editing. LR-T: Conceptualization, Methodology, Resources, Supervision, Writing – review & editing.
