# Supplementary material for: The nature of memory impairment in multiple sclerosis: understanding different patterns over the course of the disease
Source: Front Psychol. 2024 Jan 16;14:1269794. doi: 10.3389/fpsyg.2023.1269794 (PMC10828846; doi:10.3389/fpsyg.2023.1269794)
Supplement: Supplementary file 1 [file Data_Sheet_1.pdf]

*Supplementary Material*

***Supplementary Table 1. modified Auditory Verbal Learning Test results by group _ 2***
***Supplementary Table 2. Recognition trials: discriminability index and criterion level by group ____________________ 3***
***Supplementary Table 3. Univariate and multivariate linear regression with information processing speed (SDMT) and working memory (LNS) predicting the delayed free recall (m-AVLT A7) for each group____________ 4***

**Supplementary Table 1. modified Auditory Verbal Learning Test results by group**

| | m-AVLT A1 | m-AVLT A2 | m-AVLT A3 | m-AVLT A4 | m-AVLT A5 |
|---|---|---|---|---|---|
| **HC** | 6.00 (6.00 - 8.00) | 11.00 (9.00 - 12.00) | 12.00 (10.00 - 14.00) | 14.00 (11.00 - 15.00) | 13.00 (12.00 - 14.00) |
| **RIS – CIS** | 6.50 (5.00 - 9.00) | 10.50 (9.00 - 12.00) | 11.00 (10.00 - 14.50) | 13.00 (10.50 - 15.00) | 12.50 (10.50 - 15.00) |
| **RRMS** | 6.00 (5.00 - 8.00) | 8.00 (7.00 - 11.00) | 10.00 (8.00 - 12.00) | 11.00 (10.00 - 13.00) | 11.00 (9.50 - 13.00) |
| **PMS** | 5.00 (4.00 - 6.00) | 7.00 (6.00 - 8.00) | 9.00 (7.00 - 11.00) | 10.00 (8.00 - 11.50) | 10.00 (7.00 - 13.50) |

| | m-AVLT A1-A5 | m-AVLT B1 | m-AVLT A6 | m-AVLT A7 |
|---|---|---|---|---|
| **HC** | 56.00 (50.00 - 60.00) | 7.00 (6.00 - 8.00) | 12.00 (10.00 - 14.00) | 12.00 (10.00 - 14.00) |
| **CIS - RIS** | 54.00 (42.00 - 66.00) | 7.50 (6.00 - 9.00) | 11.50 (8.00 - 14.00) | 12.50 (7.25 - 14.75) |
| **RRMS** | 47.00 (41.00 - 57.00) | 6.00 (5.00 - 7.00) | 9.00 (7.00 - 12.00) | 9.00 (8.00 - 12.00) |
| **PMS** | 41.00 (32.00 - 47.50) | 5.00 (3.50 - 6.00) | 6.00 (4.00 - 10.00) | 7.00 (3.00 - 9.00) |

| | m-AVLT r1 | m-AVLT r2 | m-AVLT r3 |
|---|---|---|---|
| **HC** | 13.00 (12.00 - 14.00) | 14.00 (14.00 - 15.00) | 14.00 (13.00 - 15.00) |
| **RIS – CIS** | 12.00 (11.25 - 14.00) | 14.50 (14.00 - 15.00) | 14.00 (13.00 - 15.00) |
| **RRMS** | 13.00 (12.00 - 14.00) | 14.00 (13.00 - 15.00) | 14.00 (13.00 - 15.00) |
| **PMS** | 12.00 (10.00 - 14.00) | 14.00 (13.00 - 15.00) | 13.00 (11.00 - 14.00) |

| | m-AVLT fp1 | m-AVLT fp2 | m-AVLT fp3 |
|---|---|---|---|
| **HC** | 4.00 (2.00 - 6.00) | 1.00 (0.00 - 2.00) | 1.00 (0.00 - 3.00) |
| **RIS – CIS** | 2.50 (1.00 - 7.25) | 0.50 (0.00 - 2.00) | 1.00 (0.00 - 3.00) |
| **RRMS** | 4.00 (2.00 - 6.50) | 2.00 (0.00 - 4.00) | 2.00 (0.00 - 4.00) |
| **PMS** | 15.00 (2.00 - 9.00) | 3.00 (2.00 - 8.00) | 7.00 (2.00 - 11.00) |

Variables are presented by median (first quartile (Q1) – third quartile (Q3)).
HC, healthy controls; RIS – CIS, radiologically isolated syndrome – clinically isolated syndrome; RRMS, relapsing-remitting multiple sclerosis; PMS, progressive multiple sclerosis (secondary and primary progressive multiple sclerosis); m-AVLT, modified Auditory Verbal Learning Test; A1, list A – trial 1; A2, list A – trial 2; A3, list A – trial 3; A4, list A – trial 4; A5, list A – trial 5; A1 – A5, sum of the words of the learning trials (A1, A2, A3, A4 and A5); A7, list A – trial 7 (delayed free recall); r1, 1st recognition trial; r2, 2nd recognition trial; r3, 3rd recognition trial; fp1, false positives of 1st recognition trial; fp2, false positives of 2nd recognition trial; fp3, false positives of 3rd recognition trial.

**Supplementary Table 2. Recognition trials: discriminability index and criterion level by group**

**a) 1st Recognition trial**

| | CORRECT RECOGNITION | | | | FALSE POSITIVES | | | | | | | |
|---|---|---|---|---|---|---|---|---|---|---|---|---|
| | RATE | | Z-score | | RATE | | Z-score | | d'1 | | C1 | |
| | MEAN | SD | MEAN | SD | MEAN | SD | MEAN | SD | MEAN | SD | MEAN | SD |
| **HC** | 0.85 | 0.10 | 1.19 | 0.55 | 0.12 | 0.07 | -1.26 | 0.46 | 2.45 | 0.67 | 0.04 | 0.38 |
| **RIS - CIS** | 0.83 | 0.09 | 1.07 | 0.48 | 0.12 | 0.12 | -1.36 | 0.60 | 2.42 | 0.83 | 0.15 | 0.35 |
| **RRMS** | 0.85 | 0.12 | 1.21 | 0.61 | 0.13 | 0.10 | -1.29 | 0.57 | 2.50 | 0.75 | 0.04 | 0.46 |
| **PMS** | 0.77 | 0.14 | 0.89 | 0.58 | 0.18 | 0.16 | -1.07 | 0.65 | 1.96 | 0.74 | 0.09 | 0.50 |

**b) 2nd Recognition trial**

| | CORRECT RECOGNITION | | | | FALSE POSITIVES | | | | | | | |
|---|---|---|---|---|---|---|---|---|---|---|---|---|
| | RATE | | Z-score | | RATE | | Z-score | | d'2 | | C2 | |
| | MEAN | SD | MEAN | SD | MEAN | SD | MEAN | SD | MEAN | SD | MEAN | SD |
| **HC** | 0.94 | 0.06 | 1.77 | 0.54 | 0.04 | 0.04 | -1.88 | 0.44 | 3.64 | 0.69 | 0.05 | 0.35 |
| **RIS - CIS** | 0.94 | 0.08 | 1.79 | 0.61 | 0.05 | 0.07 | -1.89 | 0.56 | 3.69 | 0.88 | 0.05 | 0.38 |
| **RRMS** | 0.93 | 0.07 | 1.72 | 0.59 | 0.07 | 0.10 | -1.65 | 0.61 | 3.38 | 0.86 | - 0.04 | 0.42 |
| **PMS** | 0.91 | 0.09 | 1.58 | 0.63 | 0.16 | 0.17 | -1.17 | 0.70 | 2.76 | 0.79 | - 0.20 | 0.53 |

**c) 3rd Recognition trial**

| | CORRECT RECOGNITION | | | | FALSE POSITIVES | | | | | | | |
|---|---|---|---|---|---|---|---|---|---|---|---|---|
| | RATE | | Z-score | | RATE | | Z-score | | d'3 | | C3 | |
| | MEAN | SD | MEAN | SD | MEAN | SD | MEAN | SD | MEAN | SD | MEAN | SD |
| **HC** | 0.93 | 0.07 | 1.71 | 0.57 | 0.04 | 0.04 | -1.86 | 0.45 | 3.57 | 0.81 | 0.07 | 0.32 |
| **RIS - CIS** | 0.92 | 0.09 | 1.70 | 0.65 | 0.05 | 0.07 | -1.81 | 0.57 | 3.50 | 1.10 | 0.05 | 0.26 |
| **RRMS** | 0.90 | 0.11 | 1.54 | 0.69 | 0.08 | 0.09 | -1.64 | 0.60 | 3.18 | 1.11 | 0.05 | 0.33 |
| **PMS** | 0.85 | 0.12 | 1.27 | 0.69 | 0.24 | 0.21 | -0.86 | 0.77 | 2.14 | 1.11 | - 0.20 | 0.48 |

Results obtained by each group during the three recognition trails are detailed: a) 1st Recognition Trial; b) 2nd Recognition Trial; c) 3rd Recognition Trial.
HC, healthy controls; RIS – CIS, radiologically isolated syndrome – clinically isolated syndrome; RRMS, relapsing-remitting multiple sclerosis; PMS, progressive multiple sclerosis (secondary and primary progressive multiple sclerosis); Correct Recognition, correctly recognised words belonging to List A (rate and its respective Z-score are displayed); False positives, recognised words which are not included in List A (rate and its respective Z-score are displayed); d', discriminability index; C, criterion level. All variables are presented by mean and standard deviation.

**Supplementary Table 3. Univariate and multivariate linear regression with information processing speed (SDMT) and working memory (LNS) predicting the delayed free recall (m-AVLT A7) for each group**

**a) Univariate analysis for SDMT:**

| | *β* coefficient | 95% CI | p value | F* | p value* | R2 |
|---|---|---|---|---|---|---|
| HC | 0.046 | -0.022, 0.114 | 0.18 | 1.837 | 0.18 | 0.038 |
| RIS-CIS | 0.069 | -0.075, 0.213 | 0.33 | 1.012 | 0.33 | 0.053 |
| RRMS | 0.003 | -0.075, 0.081 | 0.94 | 0.006 | 0.94 | 0.000 |
| PMS | 0.189 | 0.092, 0.286 | <0.001 | 15.532 | <0.001 | 0.335 |

**b) Univariate analysis for LNS:**

| | *β* coefficient | 95% CI | p value | F* | p value* | R2 |
|---|---|---|---|---|---|---|
| HC | 0.070 | -0.228, 0.367 | 0.64 | 0.221 | 0.64 | 0.004 |
| RIS-CIS | 0.105 | -0.550, 0.760 | 0.74 | 0.114 | 0.74 | 0.006 |
| RRMS | 0.082 | -0.294, 0.458 | 0.66 | 0.190 | 0.66 | 0.003 |
| PMS | 0.612 | 0.054, 1.170 | 0.03 | 4.910 | 0.03 | 0.109 |

**c) Multivariate analysis for SDMT and LNS:**

| | Predictors | *β* coefficient | 95% CI | p value | F* | p value* | R2 |
|---|---|---|---|---|---|---|---|
| HC | SDMT | 0.045 | -0.026, 0.116 | 0.21 | 0.905 | 0.41 | 0.038 |
| | LNS | 0.017 | -0.293, 0.326 | 0.91 | | | |
| RIS-CIS | SDMT | 0.107 | -0.105, 0.319 | 0.30 | 0.628 | 0.55 | 0.069 |
| | LNS | -0.239 | -1.182, 0.705 | 0.60 | | | |
| RRMS | SDMT | -0.001 | -0.082, 0.080 | 0.98 | 0.094 | 0.91 | 0.003 |
| | LNS | 0.083 | -0.306, 0.472 | 0.67 | | | |
| PMS | SDMT | 0.163 | 0.050, 0.276 | 0.006 | 8.156 | 0.001 | 0.324 |
| | LNS | 0.277 | -0.334, 0.888 | 0.36 | | | |

Results obtained for each form of the disease: Model a) Independent variable: SDMT; Model b) Independent variable: LNS; Model c) Independent variable: SDMT and LNS.

HC, healthy controls; RIS – CIS, radiologically isolated syndrome – clinically isolated syndrome; RRMS, relapsing-remitting multiple sclerosis; PMS, progressive multiple sclerosis (secondary and primary progressive multiple sclerosis); SDMT, symbol digit modalities test (information processing speed measure); LNS, letter number sequencing (working memory measure); CI, confidence interval.

*ANOVA results
